# Análise dos Fatores Que Afetam a Trombose da Falsa Luz na Dissecção Aórtica Tipo B

**DOI:** 10.36660/abc.20250223

**Published:** 2025-08-14

**Authors:** Beatriz Vitória Carvalho Lordêlo, Álvaro Eduardo Santos Oliveira, Henri Jun Iti Okubo, Luana Macedo da Silva Nascimento, Luiz Henrique Cardoso da Silva, Pedro Pereira Tenório

**Affiliations:** 1 UNIVASF Paulo Afonso BA Brasil UNIVASF (Universidade Federal do Vale do São Francisco), Curso de Medicina, Paulo Afonso, BA – Brasil

**Keywords:** Dissecção Aórtica, Trombose, Luz

**Caro editor,**

O estudo publicado por Tang et al., intitulado *"Fatores que Afetam a Trombose da Falsa Luz na Dissecção Aórtica Tipo B"* , destacou a influência dos aspectos morfológicos da dissecção aórtica do tipo B (DATB), de acordo com a classificação de Stanford, na trombose da falsa luz previamente ao tratamento com correção endovascular da aorta torácica. Foi demonstrado que a trombose da falsa luz acontece com frequência significativamente menor em pacientes com DATB e insuficiência renal, quando comparados com aqueles com DATB com função renal normal.^1^

Do ponto de vista terapêutico, a identificação de fatores predisponentes à trombose da falsa luz pode auxiliar na personalização do tratamento. O estudo de Evangelista et al. demonstrou que a persistência de uma falsa luz patente está associada a um prognóstico desfavorável, com maior risco de complicações e necessidade de intervenções futuras.² A análise de características clínicas e morfológicas, como as realizadas por Tang et al., pode auxiliar na melhoria de estratégias terapêuticas, reduzindo a morbimortalidade e complicações graves, como ruptura e aneurismas.^1^

O estudo de Tsai et al. observou a presença de trombose parcial da falsa luz em 68% dos pacientes, a qual estava associada a um aumento de até 2,69 vezes no risco de mortalidade após a alta hospitalar. Em um período de três anos, a taxa de mortalidade desses pacientes alcançou 31,6%.^3^ A partir disso, além de abordar o impacto da relação morfológica e trombótica na aorta acometida, se mostra relevante destacar o impacto clínico da trombose parcial na mortalidade dos pacientes.

Fatores morfológicos e clínicos, como o diâmetro da aorta e a presença de insuficiência renal respectivamente, foram abordados no artigo como influenciadores da trombose da falsa luz. No entanto, fatores hemodinâmicos, como a taxa de cisalhamento e tempo de residência do fluido nos lúmens não foram relatados, os quais são críticos para formação de trombos.^4 , 5^ Estudos como o de Menichini et al.^4^ sugerem a utilização de modelo computacional para avaliar parâmetros hemodinâmicos. Dessa forma, a incorporação de simulações computacionais pode buscar associação e assim justificar como a insuficiência renal interfere em fatores hemodinâmicos e promove o desenvolvimento de trombose da falsa luz.

Parker et al. ressalta que saber a proporção da trombose do falso lúmen, definido como a razão entre o volume do trombo e o volume total do falso lúmen, é essencial para categorizar possível fator protetivo. Além disso, o estudo descartou tomografia computadorizada com falsos lúmens perfundidos inadequadamente pelo contraste, visto que impedem a modelagem aórtica e análise hemodinâmica adequadas,^6^ ao contrário do que aborda Tang et al.^1^ que considerou ausência de contraste como falso lúmen trombosado. Outrossim, se destaca a importância de ter dados sobre o estado de anticoagulação dos pacientes, pois isso afeta a presença de trombose no falso lúmen.
