# Supplementary material for: Análise dos Fatores Que Afetam a Trombose da Falsa Luz na Dissecção Aórtica Tipo B
Source: Arq Bras Cardiol. 2025 Aug 14;122(8):e20250223. [Article in Portuguese] doi: 10.36660/abc.20250223 (PMC12671605; doi:10.36660/abc.20250223)
Supplement: Translation

# Translation: Analysis of Factors Affecting False Lumen Thrombosis in Type B Aortic Dissection

**DOI:** 10.36660/abc.20250223i

**Authors:** Beatriz Vitória Carvalho Lordêlo, Álvaro Eduardo Santos Oliveira, Henri Jun Iti Okubo, Luana Macedo da Silva Nascimento, Luiz Henrique Cardoso da Silva, Pedro Pereira Tenório

**Affiliations:** 1 Paulo Afonso BA Brazil UNIVASF (Universidade Federal do Vale do São Francisco), Curso de Medicina, Paulo Afonso, BA – Brazil

**Keywords:** Aortic Dissection, Thrombosis, Light

**Dear editor,**

The study published by Tang et al., entitled *"Factors Affecting False Lumen Thrombosis in Type B Aortic Dissection,"* highlighted the influence of morphological aspects of type B aortic dissection (TBAD), according to the Stanford classification, on false lumen thrombosis prior to treatment with endovascular repair of the thoracic aorta. It has been shown that false lumen thrombosis occurs significantly less frequently in patients with TBAD and renal failure, when compared with those with TBAD and normal renal function.^1^

From a therapeutic perspective, identifying predisposing factors for false lumen thrombosis can help personalize treatment. The study by Evangelista et al. demonstrated that persistent patent false lumen is associated with a poor prognosis, with a higher risk of complications and the need for future interventions.² The analysis of clinical and morphological characteristics, such as those performed by Tang et al., can help improve therapeutic strategies, reducing morbidity and mortality and serious complications, such as rupture and aneurysms.^1^

The study by Tsai et al. observed partial thrombosis of the false lumen in 68% of patients, which was associated with a 2.69-fold increase in the risk of mortality after hospital discharge. Over a three-year period, the mortality rate in these patients reached 31.6%.^3^Therefore, in addition to addressing the impact of the morphological and thrombotic relationship in the affected aorta, it is important to highlight the clinical impact of partial thrombosis on patient mortality.

Morphological and clinical factors, such as aortic diameter and the presence of renal failure, respectively, were discussed in the article as influencing false lumen thrombosis. However, hemodynamic factors, such as shear rate and fluid residence time in the lumens, which are critical for thrombus formation, were not reported.^4 , 5^Studies such as that by Menichini et al.^4^suggest the use of a computational model to evaluate hemodynamic parameters. Thus, incorporating computational simulations can seek associations and thus explain how renal failure interferes with hemodynamic factors and promotes the development of false lumen thrombosis.

Parker et al. emphasize that knowing the proportion of false lumen thrombosis, defined as the ratio of thrombus volume to total false lumen volume, is essential for categorizing a possible protective factor. Furthermore, the study ruled out computed tomography scans with false lumens inadequately perfused with contrast, as they impede adequate aortic modeling and hemodynamic analysis,^6^contrary to what Tang et al.,^1^who considered the absence of contrast as a thrombosed false lumen, argue. Furthermore, they emphasize the importance of having data on patients’ anticoagulation status, as this affects the presence of false lumen thrombosis.
